# Effects of micro-porosity and local BMP-2 administration on bioresorption of β-TCP and new bone formation

**DOI:** 10.1186/s40824-019-0161-2

**Published:** 2019-07-26

**Authors:** Atsuhito Kakuta, Takaaki Tanaka, Masaaki Chazono, Hirokazu Komaki, Seiichiro Kitasato, Naoya Inagaki, Shoshi Akiyama, Keishi Marumo

**Affiliations:** 1 Department of Orthopaedic Surgery, Jikei University School of Medicine, 3-25-8 Nishi-shinbashi, Minato-ku, Tokyo, 105-0003 Japan; 2 Department of Orthopaedic Surgery, NHO Utsunomiya National Hospital, 2160 Shimo-Okamoto, Utsunomiya City, Tochigi 329-1193 Japan

**Keywords:** Beta-tricalcium phosphate (β-TCP), Microporous structure, Bone morphogenetic protein (BMP), Bone formation, Osteoclasts

## Abstract

**Background:**

It has been reported that the microporous structure of calcium phosphate (CaP) ceramics is important to osteoconduction. Bone morphogenetic protein-2 (BMP-2) has been shown to be a promising alternative to bone grafting and a therapeutic agent promoting bone regeneration when delivered locally. The aim of this study was to evaluate the effects of micro-porosity within beta-tricalcium phosphate (β-TCP) cylinders and local BMP-2 administration on β-TCP resorption and new bone formation.

**Methods:**

Bilateral cylindrical bone defects were created in rabbit distal femora, and the defects were filled with β-TCP. Rabbits were divided into 3 groups; defects were filled with a β-TCP cylinder with a total of approximately 60% porosity (Group A: 13.4% micro- and 46.9% macropore, Group B: 38.5% micro- and 20.3% macropore, Group C: the same micro- and macro-porosity as in group B supplemented with BMP-2). Rabbits were sacrificed 4, 8, 12, and 24 weeks postoperatively.

**Results:**

The number of TRAP-positive cells and new bone formation in group B were significantly greater than those in group A at every period. The amount of residual β-TCP in group C was less than that in group B at all time periods, resulting in significantly more new bone formation in group C at 8 and 12 weeks. The number of TRAP-positive cells in group C was maximum at 4 weeks.

**Conclusions:**

These results suggest that the amount of submicron microporous structure and local BMP-2 administration accelerated both osteoclastic resorption of β-TCP and new bone formation, probably through a coupling-like phenomenon between resorption and new bone formation.

## Background

Beta-tricalcium phosphate (β-TCP) is a calcium phosphate (CaP) ceramic used in bone grafting as an alternative bone substitute to autograft. It has been reported that β-TCP has excellent osteoconduction and resorbability when filling a bone defect [1–7]. The β-TCP block with 75% porosity that we have used can be resorbed within a few years. However, it has a compressive strength of only 3 MPa, which is inadequate for weight-bearing sites until bone incorporation occurs. Thus, we have developed a β-TCP block with 60% porosity. It has a compressive strength of 22 MPa, which is approximately seven-fold greater than that of β-TCP with 75% porosity. However, resorption of the β-TCP block with 60% porosity is relatively slow [8]. Thus, stimulation using a better microporous structure and/or growth factors is necessary to facilitate β-TCP resorption and new bone formation.

It has been reported that the microporous structure of CaP ceramics is important to osteoconduction [9–16]. The presence of micropores, by which the specific surface area of the materials is increased, seems to be essential for osteoconduction [17]. It was reported that CaP ceramics without micropores were not resorbed and there was slower bone ingrowth compared with those that had micropores [18].

Bone morphogenetic protein-2 (BMP-2) has been shown to be a promising alternative to bone grafting and a therapeutic agent promoting bone regeneration when delivered locally [19–21]. Porous CaP ceramics have been used as carriers of BMPs and have been shown to induce bone formation [22–29]. BMPs strongly induce the recruitment and differentiation of mesenchymal progenitor cells into mature osteoblasts, thereby producing a bony matrix [30]. BMPs also directly or indirectly stimulate the differentiation of osteoclast progenitor cells and promote the function of mature osteoclasts [31–34].

The aim of this study was to evaluate the effects of micro-porosity within β-TCP cylinders and local BMP-2 administration on β-TCP resorption and new bone formation.

## Methods

### β-TCP

Two types of porous β-TCP cylinders were synthesized with a mechanochemical method and provided by Olympus Terumo Biomaterials Co. (Tokyo, Japan). Briefly, CaHPO_4_2H_2_O and CaCO_3_ at a molar ratio of 2:1 were mixed into a slurry with pure water and zirconia ball in a pot mill for 24 h and dried at 80 °C. The calcium-deficient hydroxyapatite was converted to β-TCP by calcinations at 800 °C for the cylinder with 13.4% micropore, and 900 °C for the cylinder with 38.5% micropore. Following the preparation of a foaming slurry of β-TCP with a foaming agent and a drying process, the preforming porous body of β-TCP was obtained. After the preforming body was sintered at 1050 °C for 1 h, a porous β-TCP cylinder with approximately 60% porosity (38.5% micro- and 20.3% macropore) was obtained. A second type of β-TCP cylinder with approximately 60% porosity (13.4% micro- and 46.9% macropore) was obtained after sintering for 10 h. The pore size of micro- and macropores, pore distribution, and surface area were measured using a mercury porosimeter. Pores less than 10 μm in diameter were classified as micropores; pores ≥10 μm in diameter were classified as macropores [35]. The volumes of micro- and macropore were calculated from the area ratio on the scanning electronic micrographs (× 50).

### BMP-2

BMP-2 was purchased from Osteopharma Inc. (Osaka, Japan). It was Recombinant Human BMP-2 (E.coli derived). Lyophilized BMP-2 was diluted to a concentration of 5 μg/μl by adding distilled water just before implantation.

### Surgical procedure

Ninety-six New Zealand White rabbits, weighing 3.1–3.3 kg were used for the experiments. Rabbits were divided into the following 3 groups.

Group A (*n* = 8/time period) - defects were filled with β-TCP cylinder with approximately 60% porosity (60.3% total porosity; 13.4% micro- and 46.9% macropore).

Group B (*n* = 8/time period) - defects were filled with β-TCP cylinder with approximately 60% porosity (58.8% total porosity; 38.5% micro- and 20.3% macropore).

Group C (*n* = 8/time period) - defects were filled with β-TCP cylinder with the same micro- and macro-porosity as in group B, but supplemented with 12.5 μg BMP-2.

**Fig. 1:**
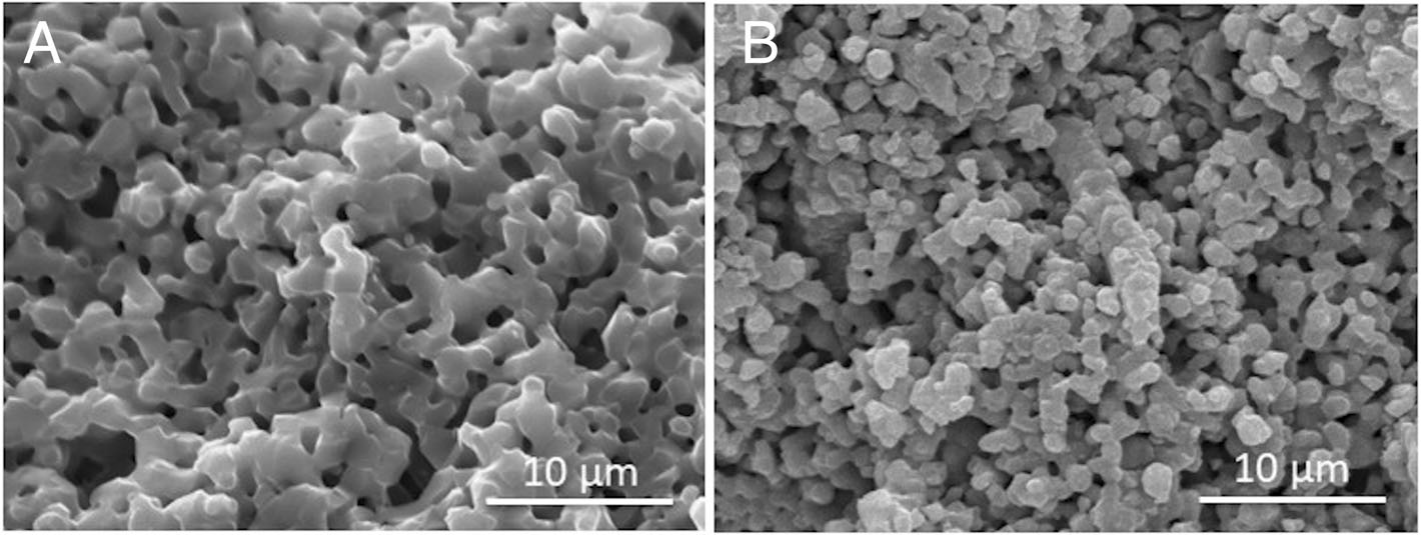
Two types of β-TCP. Scanning electron micrographs (original magnification, × 3000) of the two types of β-TCP cylinders with a total of approximately 60% porosity. 13.4% micro- and 46.9% macropore (**A**), 38.5% micro- and 20.3% macropore (**B**). The white scale bar shows 10 μm

The difference between groups A and B was the number of micropores (Fig. 1), and that between groups B and C was with or without BMP-2 administration.

**Fig. 2:**
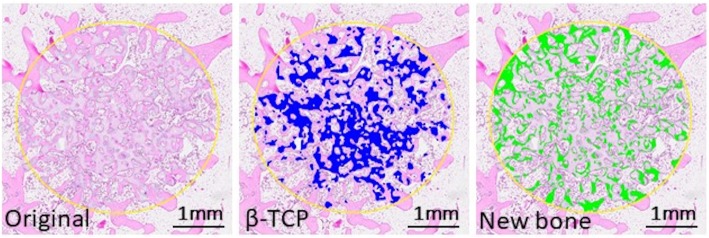
An image analysis of a bone defect**.** The surface area of newly formed bone and the remaining β-TCP was measured using an image analyzer, (WinROOF, Mitani Co., Tokyo, Japan). The colored area was used for evaluation of the residual amount of β-TCP (blue) and newly formed bone (green) in the defect. The scale bar shows 1 mm

Under intramuscular droperidol (0.25 mg/kg), intravenous pentobarbital (20 mg/kg) and general isoflurane (2%) anesthesia, bilateral cylindrical bone defects (4.1 mm W × 11 mm L) were created by drilling 1 mm anterior to the insertion of the lateral collateral ligament in the lateral aspect of the distal femur. After flushing the defects with sterile physiological saline, the hemorrhage was controlled by packing sterile gauze and the bone cavities were immediately filled with β-TCP cylinders (4.0 mm W × 10 mm L). The muscle attachment was repaired and the fascia and skin were closed in layers. After surgery, all animals were allowed to move freely in their cages without joint immobilization. Rabbits were sacrificed 4, 8, 12, and 24 weeks postoperatively, and the distal part of the femur was removed and fixed with 4% paraformaldehyde in phosphate-buffered saline. After decalcification in 0.4 M ethylenediaminetetraacetic acid for 2 weeks, serial histological sections were cut to include the deepest part of the patellar groove. Decalcified sections were obtained for hematoxylin-eosin and tartrate-resistant acid phosphatase (TRAP) staining. The surface area of newly formed bone and the remaining β-TCP was measured using an image analyzer (WinROOF, Mitani Co., Tokyo, Japan) and was expressed as a percentage of the defect (Fig. 2). Residual β-TCP and the number of TRAP-positive cells within the defects were measured to determine β-TCP resorption, whereas new bone formation was evaluated to analyze osteoconductivity.

The care and use of the animals in this study were in accordance with the guidelines of the Laboratory Animal Facilities of The Jikei University School of Medicine.

### Statistical analysis

Data were expressed as the mean ± SD. Student’s t-tests were used for pairwise comparisons with group B, for example, between groups A and B or groups B and C, for each time period separately. A *p*-value below 0.05 was considered statistically significant. The statistical analysis of the remaining amount of β-TCP between groups A and B was not performed because it was technically difficult to detect micro-pore spaces by light microscopy.

## Results

### Micro-porosity

The micropore diameter of the β-TCP block in group A (13.4% micro- and 46.9% macropore) ranged from 0.4 to 0.8 μm and that in groups B and C (38.5% micro- and 20.3% macropore) ranged from 0.7 to 1.1 μm. Most of the micropore diameter in groups B and C were submicron.

### Surface area

The surface area of the β-TCP block in group A was 0.967 m^2^/g and that in groups B and C was 1.802 m^2^/g.

### Histological characteristics

Histological examination showed that new bone formation was found in β-TCP cylinders in all groups at every period. However, β-TCP resorption, new bone formation, and number of TRAP-positive cells were different among the 3 groups.

**Fig. 3:**
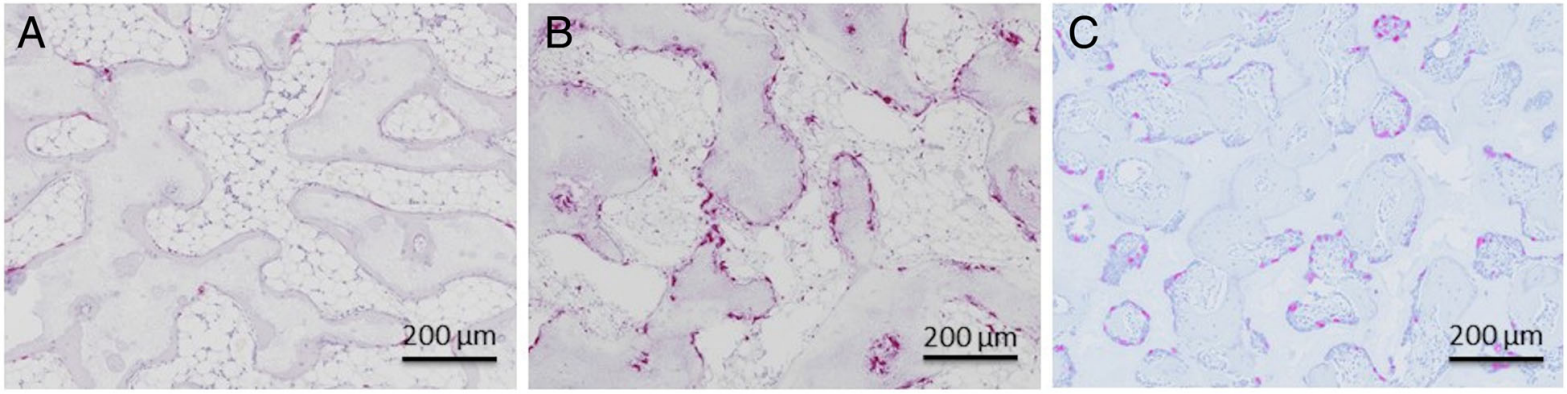
Decalcified histological sections stained with TRAP 4 weeks after implantation of β-TCP cylinders (original magnification, × 100)**. A**, **B**, and **C** indicate β-TCP cylinders in group (**A**, **B**, and **C**), respectively. TRAP-positive cells were in contact with the surface of the β-TCP cylinders in all groups at 4 weeks, but the number of TRAP-positive cells in group **A** was significantly smaller than that in groups (**B** and **C**). The scale bar shows 200 μm

TRAP-positive cells were in contact with the surface of β-TCP cylinders in all groups at 4 weeks, but the number of TRAP-positive cells in group A was smaller than that in groups B and C at 4 weeks (Fig. 3). β-TCP resorption and new bone formation occurred at the periphery of the β-TCP cylinders at 4 weeks in all groups and progressed toward the center of the β-TCP cylinders in a time dependent manner in all groups.

**Fig. 4:**
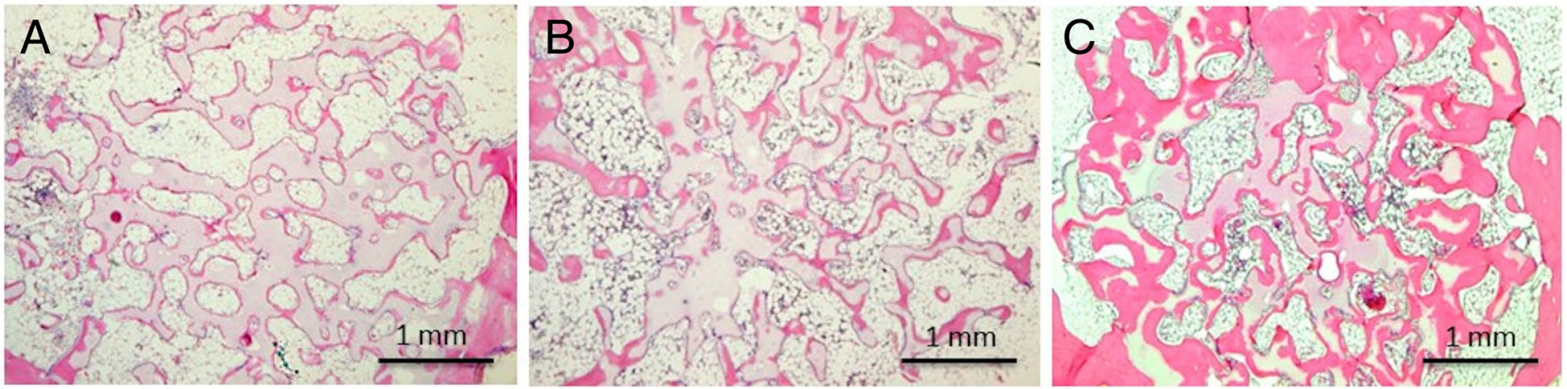
Decalcified histological sections stained with hematoxylin-eosin 12 weeks after implantation of β-TCP cylinders (original magnification, × 40). **A**, **B**, and **C** indicate β-TCP cylinders in group **A**, **B**, and **C**, respectively. New bone formation was found in β-TCP cylinders of all groups at 12 weeks. The new bone in group **A** was thinner than that in group **B**, and that in group **C** was thicker than that in group **B**, assessed qualitatively. The scale bar shows 1 mm

New bone formation was found in β-TCP cylinders of all groups by 12 weeks, but the new bone in group A was thinner than that in group B, whereas that in group C was thicker than that in group B, assessed qualitatively (Fig. 4).

After 24 weeks, new bone formation was found in the center of the β-TCP cylinders and the amount of new bone was greater than that at 4 weeks in all groups. Most of the β-TCP in group C was resorbed.

### Comparison of group A with B

**Fig. 5:**
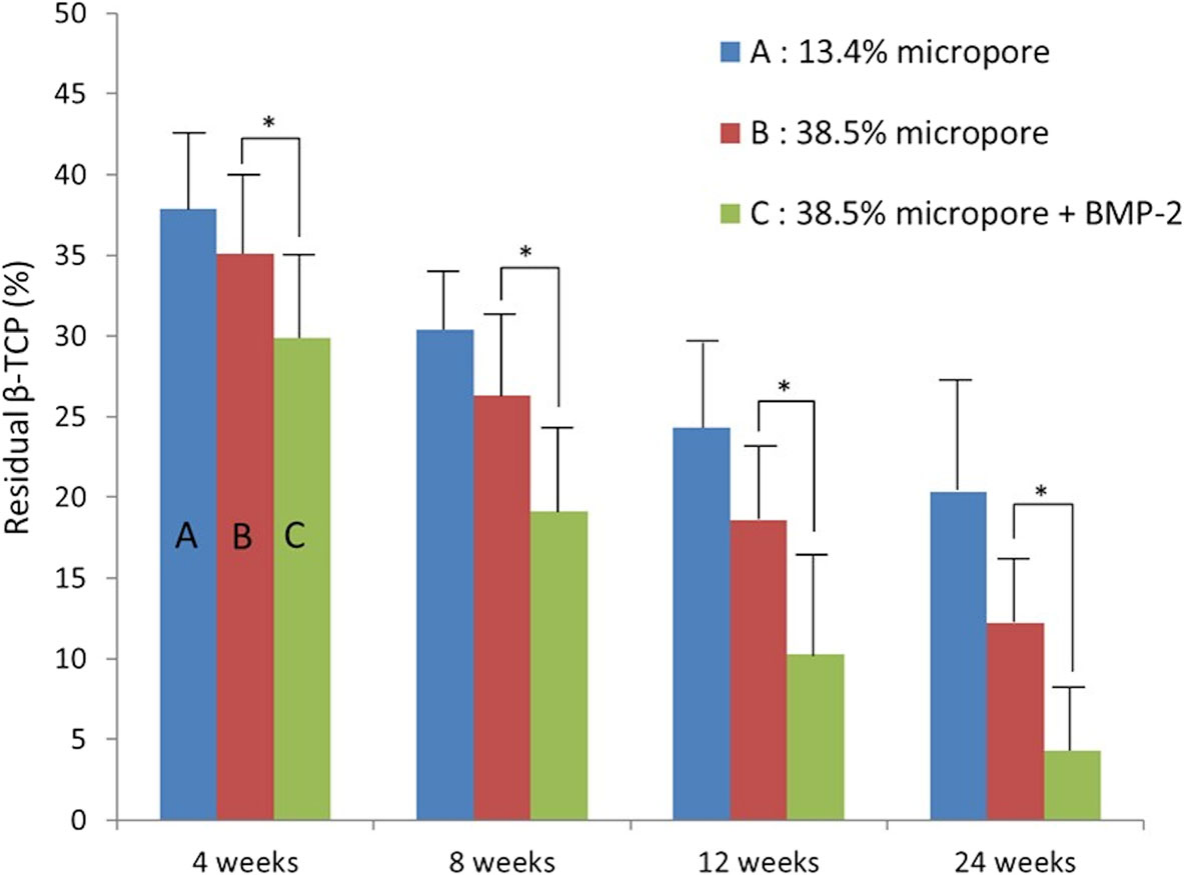
Residual β-TCP. The residual amount of β-TCP in group A was greater than that in group B at every period, but statistical significance could not be demonstrated. The residual amount of β-TCP in group C was significantly lower at every period compared to that in group B (*p < 0.05)

**Fig. 6:**
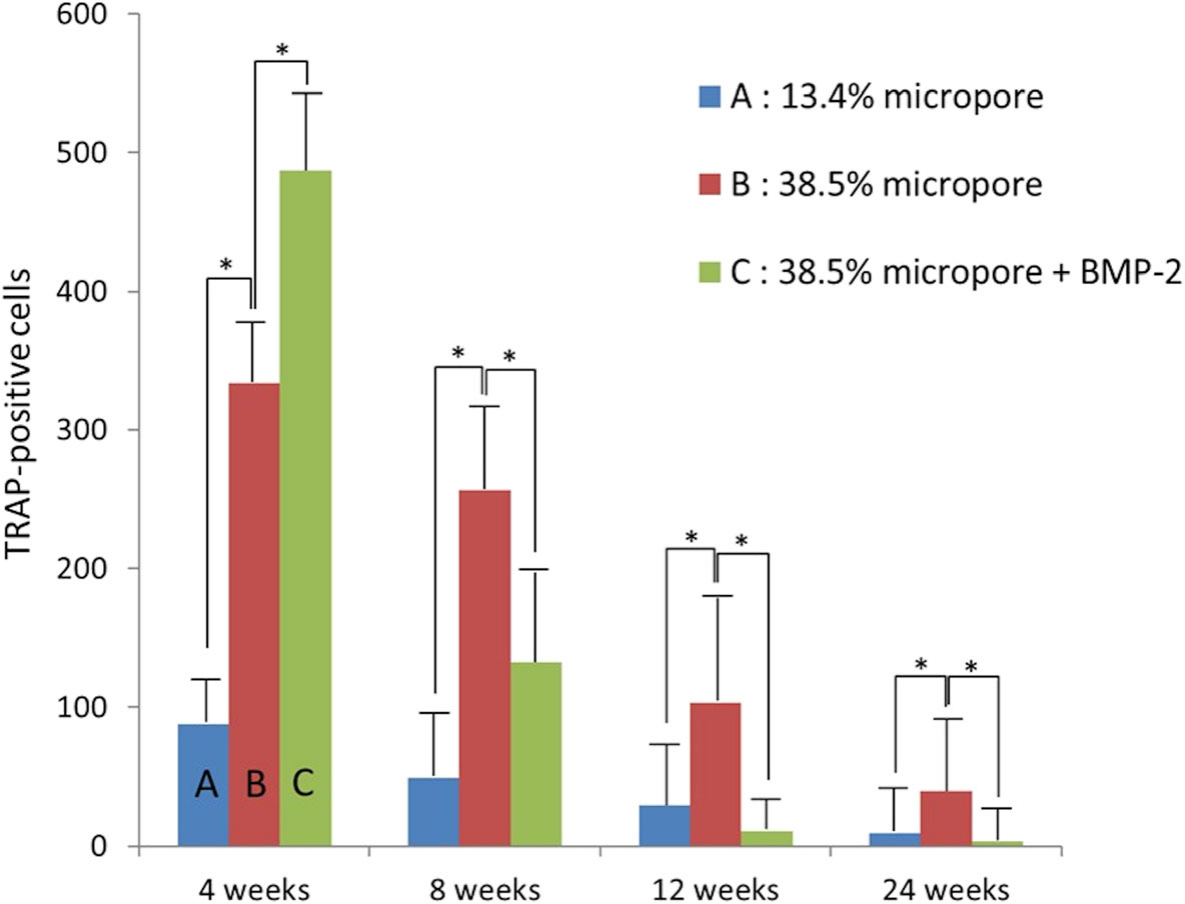
TRAP-positive cells**.** The mean number of TRAP-positive cells in group A was significantly smaller than that in group B at every period. The mean number of TRAP-positive cells in group C was larger than that in group B at 4 weeks, but smaller than that in group B at 8, 12, and 24 weeks (*p < 0.05)

**Fig. 7:**
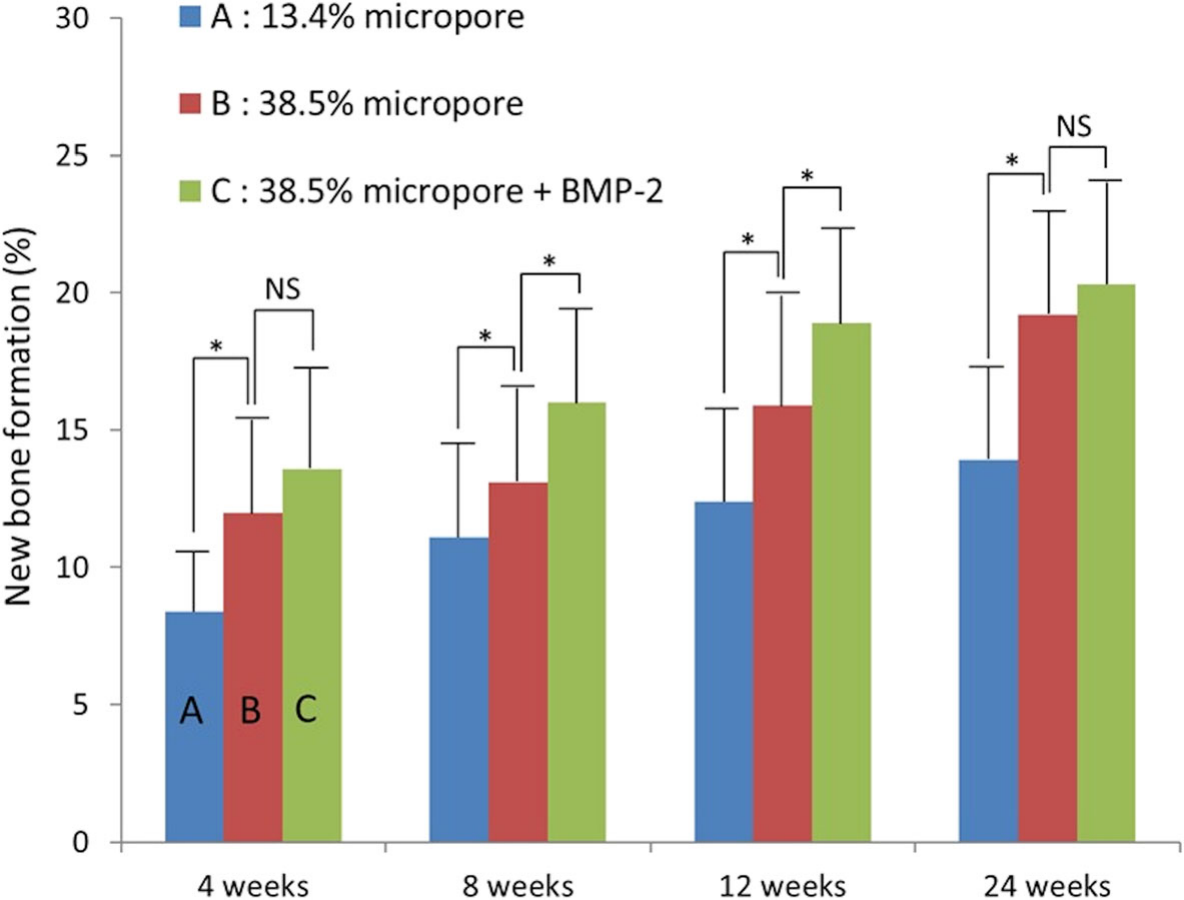
New bone formation**.** New bone formation in group A was significantly less than that in group B at every period. The new bone formation in group C was significantly greater than that in group B at 8 and 12 weeks (*p < 0.05). NS (not significant)

The mean amount of residual β-TCP in group A at 4, 8, 12, and 24 weeks was 37.9, 30.4, 24.3, and 20.3%, whereas that in group B was 35.1, 26.3, 18.6, and 12.2%, respectively (Fig. 5). The residual amount of β-TCP in group A was greater than that in group B at every period, but statistical significance could not be demonstrated because of the technical difficulties in detecting micro-pore spaces by light microscopy. The mean number of TRAP-positive cells in group A at 4, 8, 12, and 24 weeks was 87.8, 49.1, 29.4, and 9.4, whereas that in group B was 333.8, 256.8, 103.2, and 39.4, respectively (Fig. 6). The mean number of TRAP-positive cells in group A was significantly smaller than that in group B at every period. The mean amount of new bone formation in group A at 4, 8, 12, and 24 weeks was 8.4, 11.1, 12.4, and 13.9%, whereas that in group B was 12.1, 13.1, 15.9, and 19.2%, respectively (Fig. 7). New bone formation in group A was significantly less than that in group B at every period.

### Comparison of group B with C

The mean amount of residual β-TCP in group C at 4, 8, 12, and 24 weeks was 29.9, 19.1, 10.3, and 4.3%, respectively (Fig. 5). The residual amount of β-TCP in group C was significantly less than that in group B at every period. The residual amount of β-TCP in group B was 12.2% at 24 weeks, whereas most of the β-TCP in group C was resorbed by 24 weeks. The mean number of TRAP-positive cells in group C at 4, 8, 12, and 24 weeks was 486.9, 132.5, 10.6, and 3.3, respectively (Fig. 6). The mean number of TRAP-positive cells in group C reached a maximum and was larger than that in group B at 4 weeks, but significantly smaller than that in group B at 8, 12, and 24 weeks. The mean new bone formation in group C at 4, 8, 12, and 24 weeks was 13.6, 15.9, 18.9, and 20.3%, respectively (Fig. 7). New bone formation in group C was greater than that in group B at every period. However, statistical significance was found only at 8 and 12 weeks.

## Discussion

### Material resorption

De Groot suggested that the rate of degradation was determined by implant micro-porosity [36]. Macroporous materials without micro-porosity allow only bone ingrowth, whereas dense materials without pores of either type showed little degradation [37].

In the present study, the amount of micropores in group A was approximately 1/3 of that in group B. Despite the similarity in total porosity in groups A and B, β-TCP resorption was very different. The mean amount of residual β-TCP in group A and that in group B at 4 weeks was almost equal. Resorption of β-TCP in both groups occurred in a time dependent manner through 24 weeks. However, the mean amount of residual β-TCP in group B was less than that in group A at 24 weeks.

β-TCP resorption is thought to involve both solution- and cell-mediated disintegration. In our previous animal experiments, we have found numerous multinucleated giant cells on the surface of the β-TCP; most of these cells were positively stained for TRAP [4]. In the present study, both the number of TRAP positive cells and the residual amount of β-TCP in groups A and B decreased in a time dependent manner. However, the number of TRAP positive cells in group B was significantly higher than that in group A at every period, likely responsible for the lower amount of residual β-TCP in group B through 24 weeks. Thus, it is hypothesized that the number of TRAP positive cells resulting in β-TCP resorption is affected by the amount of micro-porosity.

Recently, Davison reported that in vitro, the submicron-scale surface structure of TCPs promoted osteoclast-like cell activity, fusion, and secretion of factors that amplified osteogenic differentiation of human mesenchymal stem cells when compared to micron-scale topography. Their in vivo study also showed that TCPs possessing submicron grains, pores, and roughness promoted extensive osteoclast-like cell colonization and considerable ectopic bone formation. Multinucleated osteoclast-like cells (OCLC) were observed on a submicron-scale in TCP material, but there were no OCLC on a micron-scale in TCP material [38]. In the present study, most of the micropore diameters of the β-TCP used in all groups were submicron. Our previous electron microscopic studies showed that multinucleated giant cells were in contact with the surface of β-TCP at an early stage following implantation of β-TCP. Some of them had a ruffled border at the cell-substrate interface, characteristic of osteoclasts [39].

These findings indicate that a microporous structure could provide a better microenvironment for osteoclasts, resulting in acceleration of resorption of β-TCP.

### New bone formation

Some argue that micropore connection size is a critical factor with small connection sizes resulting in lower rates of bone formation [40]. Others report that the microstructure increases surface roughness, which, in turn, increases attachment, proliferation and cell differentiation [10, 41]. In the present study, new bone formation in group B was significantly greater than that in group A at every period. Furthermore, new bone in group B assessed qualitatively was thicker than that in group A.

Davison reported that in vivo, the submicron-scale surface structure of TCPs induced greater bone formation in the free implant space whereas micron-scale TCPs did not form bone in any of the subjects [38]. Many studies have shown that the specific surface area of the scaffold can be enhanced by increasing micro-porosity, thereby providing more protein adsorption sites (e.g. BMPs) [42–46]. Osteoinductive proteins can subsequently stimulate the osteogenic-related functions of cells, such as attachment, proliferation, osteogenic differentiation and biomineralization. Our previous studies showed that most of the collagen fibrils were located within micropores at an early stage following implantation of β-TCP [39]. These findings suggest that micropores may provide an environment for collagen formation, leading to the deposition of apatite crystals.

In the present study, new bone formation was found on the periphery of β-TCP cylinders at 4 weeks and proceeded toward the center by 24 weeks through increased cell invasion and blood flow from the periphery. In our previous study using β-TCP cylinders with 75% porosity, new bone formation peaked at 4 weeks and decreased thereafter [4]. In contrast, in the present study using 60% porosity β-TCP cylinders, new bone formation increased monotonically through 24 weeks. The difference in the peak time for new bone formation between cylinders with 75% or 60% porosity may be correlated to the residual amount of β-TCP and the number of TRAP positive cells. Most β-TCP cylinders with 75% porosity was resorbed and replaced by bone by 12 weeks. In contrast, 12.2% of the β-TCP with 60% porosity in group B still remained even 24 weeks after implantation. These findings indicate that the micropores could facilitate β-TCP resorption and new bone formation. In the context of bone remodeling, osteoclasts contribute to bone formation to communicate with osteoblasts in a crosstalk that regulates the local recruitment and bone forming activity of osteoblasts. Thus, osteoclast-mediated resorption may play an important role in bone formation in the β-TCP implanted area.

### BMP-2 administration

The residual amount of β-TCP in group C was significantly less than that in group B at every period and decreased in a time dependent manner in both groups. However, the number of TRAP-positive cells in group C was significantly larger than that in group B at 4 weeks, but significantly smaller than that in group B at 8, 12, and 24 weeks. Few TRAP-positive cells were found in group C at 12 and 24 weeks. BMPs not only induced bone formation, but also stimulated osteoclastogenesis and more rapid bone resorption [47–49]. Thus, the fact that the number of TRAP-positive cells in group B was larger than that in group C at 8 weeks and thereafter occurred because the bone resorptive effects of BMP-2 were accelerated at an early stage. New bone formation in group C was greater than that in group B at every period, and significantly greater at 8 and 12 weeks. These results suggest that BMP-2 accelerated β-TCP resorption and replacement by bone in the presence of submicron micropores. Our previous clinical studies showed that approximately 1/3 of the β-TCP block with 60% porosity remained 6 years after performing opening high tibial osteotomy (HTO) [50]. The β-TCP cylinders with 60% porosity used in the present study were very similar to those in clinical use. Thus, the present study may allow safer and earlier weight-bearing in patients after opening HTO.

## Conclusion

Three β-TCP cylinders with different micro-porosities or with BMP-2 administration were compared in terms of material resorption and new bone formation using rabbit cancellous bone defects over 24 weeks. The results showed that more submicron microporous structure facilitated osteoclastic resorption of β-TCP and new bone formation. In addition, local BMP-2 administration further accelerated both osteoclastic resorption of β-TCP and new bone formation. A combination of β-TCP with superior microstructure and local BMP-2 administration could accelerate bone reconstruction.

## Data Availability

All data generated or analyzed in this study are included in this published article.

## Abbreviations

BMP-2: Bone morphogenetic protein-2
CaP: Calcium phosphate
HTO: High tibial osteotomy
OCLC: Osteoclast-like cells
TRAP: Tartrate-resistant acid phosphatase
β-TCP: Beta-tricalcium phosphate
